# Comparison among the efficacy of interventions for the return rate to receive the pap test report: randomized controlled clinical trial^1^

**DOI:** 10.1590/1518-8345.1337.2857

**Published:** 2017-03-09

**Authors:** Camila Teixeira Moreira Vasconcelos, Ana Karina Bezerra Pinheiro, Ana Izabel Oliveira Nicolau, Thaís Marques Lima, Denise de Fátima Fernandes Barbosa

**Affiliations:** 2 PhD, Adjunct Professor, Departamento de Enfermagem, Universidade Federal do Ceará, Fortaleza, CE, Brazil.; 3 PhD, Associate Professor, Departamento de Enfermagem, Universidade Federal do Ceará, Fortaleza, CE, Brazil.; 4 PhD, Adjunct Professor, Centro Universitário Estácio, Fortaleza, CE, Brazil.; 5 MSc, Professor, Departamento de Enfermagem, Universidade Federal do Ceará, Fortaleza, CE, Brazil.

**Keywords:** Intervention Studies, Papanicolaou Test, Uterine Cervical Neoplasms, Nursing, Health Education

## Abstract

**Objective:**

to test the effects of a behavioral, an educative and a comparative intervention on women's adherence to the return appointment to receive the pap test report.

**Methods:**

randomized controlled clinical trial at a Primary Health Care Service, involving three groups: EG (educative session and test demonstration), BG (recall ribbon) and standard intervention (card containing the return appointment - graphical reminder), called comparative group here (CG). To select the sample, the following was established: having started sexual activity and undergoing the pap smear during the study, resulting in 775 women.

**Results:**

among the 775 women, 585 (75.5%) returned to receive the test result within 65 days. The educative group presented the highest return rate (EG=82%/CG=77%/BG=66%), statistically significant only when compared to the behavioral group (p=0.000). The educative group obtained the smallest interval (p<0.05) concerning the mean number of days of return to receive the test result (EG:M=43days/BG:M=47.5days/CG:M=44.8 days).

**Conclusion:**

the educative group reached higher return rates and the women returned earlier, but the behavioral intervention showed to be the least effective. Brazilian Clinical Trial Register: RBR-93ykhs.

## Introduction

In Brazil, the oncologic colpocytology, also known as the Pap smear, is the reference screening method for cervical cancer, being a low-cost and simple method, applied by highly trained professionals, with a high preventive potential. The factors related to the low preventive impact of the test, however, include the late use of the health services among the women at risk. Other motives are the lack of monitoring and adequate treatment for all women screened^1^.

Besides the difficulties to adhere to the screening, the lack of follow-up is an extremely relevant factor, considering that, when there is no adequate monitoring, the entire financial investment made to perform and analyze the test is interrupted. One of the aspects that contribute to the lack of adequate follow-up of the patients screened is that the woman does not return to receive the test result. This situation is complex and multifactorial, entailing a high cost for society, as the final impact of the financial and professional investment in each test performed is negatively affected^2^.

The problem of not returning is present in different contexts^2^ ^-^ ^4^, although to varying extents, indicating the need for the health professionals responsible for preventing Cervical Cancer (CC) to use cognitive strategies, aiming to enhance the women's knowledge on the control of this disease, emphasizing the importance of the return appointment; as well as behavioral strategies, aiming to strengthen the return behavior, using reminders (graphical, visual or phone calls); and social strategies, which can be changes in the current health system, aiming to enhance the dynamics of care to bring down these figures.

A review involving the theme demonstrates that countless interventions have been tested in the fight against CC, although emphasizing women's increased adherence to the pap smear, but without any record of interventions to reduce the non-return rates for follow-up^5^. In addition, another factor that motivated this research was the finding, in a preliminary study, undertaken at a Primary Health Care Service (PHCS) in Fortaleza-CE, of the high rate (24%) of Pap test results the users did not receive^2^.

Hence, the objective in this study is to test the effects of a behavioral, an educative and another comparative intervention on women's adherence to the return appointment to receive the Pap test result. This kind of studies are relevant as they intend to propose and assess the use of interventions to minimize the absenteeism rate from the return appointment to receive the Pap test result, thus improving women's health care, reducing unnecessary spending for the health system and stimulating nurses to undertake strategies to intervene in the problems related to CC control.

## Method

Randomized controlled clinical trial involving women who were waiting to undergo the Pap test at a Primary Health Care Service (PHCS) located on the outskirts of Fortaleza, state of Ceará, in the Brazilian Northeast. The service chosen presented a high non-return rate (24%) according to a preliminary study^2^.

All women who had started sexual activity and underwent the CC prevention test at the PHCS during the data collection period were considered eligible. Women with cognitive limitations that prevented answering the questionnaire or participating in the educative or behavioral intervention and not having undergone the pap smear for any reason were excluded from the sample.

Based on the formula for studies with comparative groups and the addition of 10% for any losses, 233 participants would be needed in each group, totaling 699 women.

The outcome to be tested is the rate of non-return to receive the pap test result after receiving any of the interventions proposed in the study. Hence, the women were randomly allocated to three groups:

- Group 1 - Comparison: only the return appointment was offered to the women (card containing the data, the name of the professional and the time).

- Group 2 - Educative Intervention: besides the return appointment (card containing the date, the name of the professional and the time), an educative intervention was offered to the women.

The educative intervention was based on Freire's premises and happened in two phases^6^: problem-situations and demonstration of the Pap test. Both phases took place during a single meeting, with the number of women who attended to undergo the Pap test (maximum ten) during the period proposed for the educative intervention. The responsible researcher applied all educative interventions at the meeting room of the service.

Five graphical representations or illustrations (figures) were used for discussion. For the demonstration phase of the Pap smear, an anatomic model of the female pelvis and the material used during the Pap test were used.

- Group 3 - Behavioral Intervention: a recall ribbon was offered for the patients to put on their wrist, on which the following words were printed: return date (__/__/__), time (__:__) and the name of the professional. The dates and times were written in their respective spaces with a pen appropriate for textile.

The behavioral intervention is based on Skinner's concepts, which defined using any stimulus to strengthen the occurrence of a behavior^7^, which was the recall ribbon in this case, used to increase the women's return rate to receive the test result. The value of the reinforcement depends on its meaning for a certain individual, and the same reinforcement can affect people in different ways.

Among the different behavioral interventions, this was chosen because it is cheap and because using this kind of ribbon was a regional habit at the time of the research. The ribbons used are called recall ribbons, made of 100% polyester, 10 mm wide, ordered in three different colors (blue, pink and purple) to facilitate the women's acceptance.

As the CC prevention appointments at the institution were not scheduled, the systematic randomization used was based on the average number of care sessions per month to define the intervention (behavioral and educative) and comparative groups. This scheme was also chosen to impede the information exchange among the patients awaiting the test on that day, and with patients who undergo the CC prevention test but attend the health service daily for other care, in rooms where the patients awaiting the Pap smear are also waiting. The intention was to impede the possible contamination (information exchange) among women who received and did not receive the educative or behavioral intervention.

Thus, during a total period of six months of data collection, the groups were changed at every 60 consecutive days. During the first 60 days, the data from the comparative group were collected, followed by the behavioral group and finishing with the educative group.

In this research, due to the small number of participating researchers, the duration of the data collection (six months) and the large sample, only the professionals (employees of the institution) who performed the Pap smears were blinded because not only did they not know the timetable and the arrangement of the groups, but the respective interventions were performed before the patients entered the consultation room to undergo the test. That guaranteed that all patients were equally exposed to the possible individual orientations provided during the consultations.

**Figure 1:**
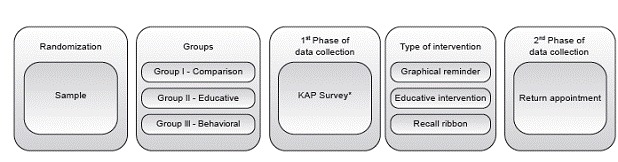
Data collection flowchart

The data were collected at two times, described according to the flowchart below (Figure 1):

The data collection instrument on the identification and the validated Knowledge, Attitude and Practice (KAP) assessment survey^8^ was applied to all women in the sample when they were awaiting the appointment, in one of the consultation rooms available at the health service.

Besides the sociodemographic variables, the assessment using the KAP is justified to measure if the groups consisted of women with a similar profile concerning these variables, which could influence the main outcome.

To guarantee the right to a return appointment equally to the women, all of them had an appointment scheduled with the primary researcher within 55 days after the test.

One of the researchers scheduled the return appointment in an agenda after the initial data collection and marked it on a card (comparative and educative intervention group) or on the recall ribbon (behavioral group), which the patient received. All patients were informed that, if they were unable to attend at the date scheduled, they could return on any date the Pap smear was being collected at the service.

For statistical ends, a deadline of up to 65 days was established to receive the result. Thus, all calculations were made considering those women who attended within 65 days to receive the test result as "returned" and, even if the tests were delivered to the patients after that period, they were classified as "did not return".

On the day of the return appointment, the instrument data were collected on the assessment of the return appointment and on the result of the Pap smear. The interventions applied were assessed by verifying all women who had returned to receive the test result.

The collection period of the KAP survey (1^st^ phase) went from September 2010 till February 2011, totaling six months. As the return appointment (2^nd^ phase) extended up to 65 days after the test, however, the total collection period went until the middle of May 2011 (approximately nine months).

The data were compiled and analyzed using the software *Statistical Package for the Social Sciences* (SPSS), version 20.0. The continuous variables were expressed as means ± standard deviation with a 95% confidence interval (CI), and the categorical variables as frequencies and percentages.

To assess the effects of the intervention on the return rates, initially, the groups were assessed for the homogeneity of the sample, concerning the identification data, the knowledge, attitude and practice of the Pap smear present in the KAP survey. Differences of means for age, years of study (education) and the onset of sexual activity between the research groups were calculated using the variance analysis test (ANOVA). For the categorical variables, Pearson's chi-square test was used for intergroup comparison. Next, two parameters were considered to assess the intervention effects: intergroup comparison of the percentage of women who returned by means of Pearson's Chi-square test; intergroup comparison of the mean number (±SD) of days for the patients to attend the return appointment by means of variance analysis (ANOVA) with Bonferroni's correction.

The clinical trial was described according to the recommendation of the international guide CONSORT (Consolidated Standards of Reporting Trials) for non-pharmacological interventions^9^. Approval for the research was obtained from the Research Ethics Committee at Universidade Federal do Ceará under protocol 81/09. The clinical trial was included in the Brazilian Clinical Trial Register under number: RBR-93ykhs.

## Results

**Figure 2:**
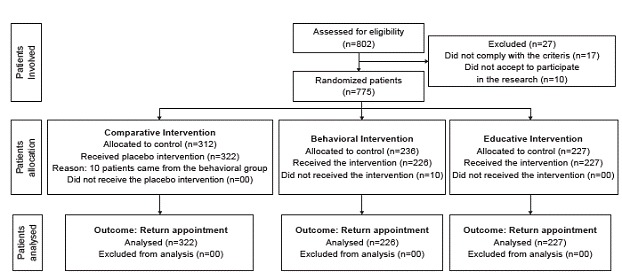
Flowchart of research development

During the research, some sporadic campaigns were held to take the Pap smear and other women's health activities, which was not previewed and which implied a larger number of women than the sample calculation who were initially included (n=802) in the total sample and per group. Nevertheless, 27 were excluded, totaling 775 patients (Figure 2). Ten women from the behavioral group did not accept to use the recall ribbon and were automatically included in the comparative group.

The comparison of the variables listed evidenced that the groups are statistically comparable (Table 1). Thus, none of these factors influenced the return rate to receive the Pap test result measured between the groups, confirming the efficacy of the randomization.

**Table 1:** Intergroup comparison according to baseline variables. Fortaleza, CE, Brazil, 2011

Variable	Comparative Group (n=322/41.5%) M*±SD**^†^** (CI**^‡^** 95%)	Educative Group (n=227/29.3%) M*±SD**^†^** (CI**^‡^** 95%)	Behavioral Group (n=226/29.2%) M*±SD**^†^** (CI**^‡^** 95%)	Test F**^§^** p**^||^**
Age (years)	35.3±14.5(33.4-37.1)	36.7±13.7(33.3-40.0)	34.8±12.7 (33.1-36.5)	0.450 0.638
Education (years)	7.1±3.8 (6.6 -7.5)	7.1±3.8 (6.2 - 8.1)	7.0 ±4.1 (6.4 - 7.6)	0.019 0.982
Onset of sexual activity (years)	16.9 ±3.6 (16.4-17.3)	16.6±2.8(15.9-17.3)	16.5±3.3 (16.0-17.0)	0.680 0.507
	N %	N %	N %	χ**^2¶^** p**^||^**
Marital status				1.131 0.56
No partner	103 32.0	71 31.3	63 27.9	
With partner	219 68.0	156 68.7	163 72.1	
Paid job				1.022 0.60
Yes	122 37.9	80 35.2	90 39.8	
No	200 62.1	147 64.8	136 60.2	
Lives nearby				1.799 0.40
Yes	299 92.9	216 95.2	215 95.1	
No	23 7.1	11 4.8	11 4.9	
Inappropriate knowledge	234 72.7	155 68.3	171 75.7	3.125 0.21
Inappropriate attitude	201 62.4	152 67.0	138 61.1	1.903 0.38
Inappropriate practice	100 31.1	70 30.8	69 30.5	0.017 0.99
Did not return during latest test	19 6.6	10 5.0	18 8.9	2.547 0.28

M*= Mean SD†= Standard Deviation CI‡= Confidence Interval F§= ANOVA p||= Significance level χ2¶ = Chi-squared

During the research, it was guaranteed that all patients would have a return appointment scheduled with the research within an average 43 (±5.6) days. Although the interval to schedule the interventions went as planned (<65 days), there was a difference among the groups, even if unintentional, concerning the mean number of days between the tests and the return appointment date (Comparative group: M=42.7/95%CI=42.0-43.3, Educative Group: M=41.4/95%CI=40.6-42.3, Behavioral Group: M=45.4/95%CI=44.9-45.9), which was due to the fact that, in some months, there were more holidays than in others, making it difficult to schedule the appointments at shorter intervals.

**Figure 3:**
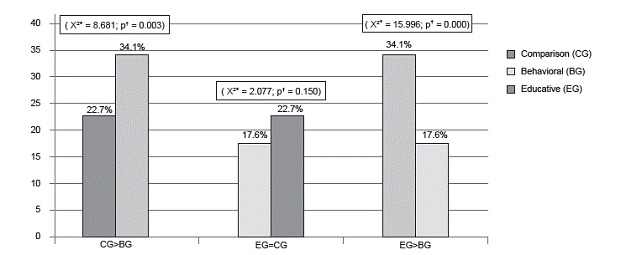
Comparison among percentages of non-attendance to the return appointment. Fortaleza, CE, Brazil, 2011

Among the 775 women in the sample, the majority (n=643/83%) attended to receive the test result, although approximately 28% (n=181) did so after the return appointment date, and were therefore automatically considered as "not returned". Out of the 775 women who underwent the test, 585 (75.5%) returned to receive the test result within 65 days (Comparative group: n=249/77.3%, Educative Group: 187/82.4%, Behavioral Group: 149/65.9%). When the non-return rates were compared among the groups, both the educative and the comparative group obtained lower rates than the behavioral group (p<0.05) (Figure 3).

The behavioral group received a statistically higher percentage of non-return than the educative and comparative groups. In addition, the risk of non-return for women in the behavioral group was 2.4 times (95%CI:1.5-3.7) that of women in the educative group; and 1.7 (95%CI:1.2-2.5) times that of women in the comparative group.

On the other hand, when comparing the mean number of days for the women to attend to receive the test result, it is clear that the educative group returned within the shortest interval (Table 2).

**Table 2:** Distribution of women who returned according to mean number of days between the test and the return appointment. Fortaleza, CE, Brazil, 2011

Group	N	M (days)*	SD**^†^**	CI**^‡^** (95%)
Comparative (CG)	249	44,8**^||^**	7,4	43,9 - 45,8
Educative (EG)	187	43,0**^||^**	7,1	42,0 - 44,0
Behavioral (BG)	149	47,5**^||^**	5,5	46,6 - 48,4
Total	585	44,9	7,1	44,4 - 45,5

M*= Mean SD†= Standard Deviation CI‡= Confidence Interval F§(ANOVA) = 17.793 (p = 0.000) || Bonferroni Post-Hoc: (CG > EG= 0.020), (BG >CG= 0.001), (BG >EG= 0.000)

## Discussion

Learning is defined as a change of behavior (knowledge, attitudes and/or skills) that can be observed or measured and that occurs at any time or place as a result of the exposure to an environmental stimulus. Specifically, patient education is the process of helping people to learn health-related behaviors that can be incorporated into the daily life with a view to optimizing health and independence in self-care. In this context, an interactive approach of partnership grants the clients the opportunity to explore and expand their skills, so that they can understand and experience the process more autonomously^10^.

The educative intervention tested in this research was not intended to measure the knowledge gained after the intervention, but to assess the influence of this knowledge on the women's behavior concerning attendance to the return appointment. Although the proportion of patients in the comparative group who returned was high (77%), the data revealed that the educative intervention reached higher proportions (82%) and, mainly, that it was able to make the women return earlier, being the group with the lowest mean number of days to attend the return appointment, confirming the power of education to improve self-care.

The fact that the woman returned earlier indicates that she understands the importance of the return appointment (theme addressed during the intervention), as well as the benefits of acting early to avoid advanced cervical cancer. It also allows the professional to improve the care, solving possible problems that might be present.

Most educative/cognitive interventions related to the early detection of CC revealed that static strategies like sending letters, pamphlets or videos, using simple language, addressing aspects related to the Pap smear, positively affected the knowledge, although this did not mean an improvement in the practice of the test^5^.

In that perspective, the educative strategies and technologies in the health service routines need to improved in order to positively affect the knowledge and attitude, but especially the healthy practice, which is more challenging for health managers and professionals. In Brazil, where most of the population still presents a low education and income level, the educative practices gain greater importance in the transformation and adoption of health behaviors, thus highlighting the value of singular informative and educative projects, which respect the needs and limitations of the users.

It seems that, when the professional offers the information with room for specific questions on the test, or even to talk about the related fears, the procedure rates increase when compared to static information supply only (letters, pamphlets, videos, etc.)^5^ ^,^ ^11^ ^-^ ^12^.

The knowledge on the benefits of CC screening, the care needed to accomplish a high-quality test and the importance of the return appointment influenced the tested women's behavior. In addition, as the behavior predicts the practice, higher return rates can be expected over time, as informed clients adhere more easily to the health professionals' recommendations, find innovative ways to cope with the disease and are less susceptible to complications. Mainly, they get more satisfied with the care when they receive adequate information on how to take care of themselves^13^.

As opposed to the cognitive theorists, the behavioral theorists sustain that the use of reinforcement is fundamental to enhance or reduce behaviors in each individual, taking into account antecedent stimuli, but mainly consequent (reinforcing) stimuli^14^. In this research, the women in the behavioral group obtained the lowest proportions of attendance to the return appointment and the patients who attended did so in a longer interval.

Among the disadvantages of using behavioral interventions is the fact that the behavioral change tends to worsen over time, especially when the clients return to their natural environment^15^. This particularity may have been responsible for the lower rates of return in the group that received the behavioral intervention, as the expected behavior should take place within 65 days.

Some women from the behavioral group who attended the return appointment were not wearing the ribbon. When asked, they revealed the following justifications: afraid that the ribbon would tear; the ribbon tore before the return date (laundresses); constraint due to the possibility of being questioned in public about the purpose of the ribbon; not wanting the ribbon visible on an important occasion. In that sense, for the sake of an effective reinforcement, it should be analyzed what types of reinforcement can enhance or reduce behaviors in each individual, strengthening the extrinsic or intrinsic motivation^14^. In this research, the recall ribbon was the least effective to reduce the patients' rates of non-return to receive the test result. Nevertheless, the comparative group, which only received a return card, which can be considered a type of graphic reminder, revealed to be a better strategy.

Independently of the work environment, the health professionals deal with a complex variety of people, who can benefit, concerning the learning, from the use of a certain type of intervention, strengthening the need to combine strategies. In addition, the preferred forms of learning and processing of each person can help to determine the selection of the most appropriate theoretical approaches, that is, considering that some learn by action and response (behaviorist), while others can learn through perception and thinking (cognitive) ^16^.

The most relevant contribution of this research is that it evidences that strategies, mainly those that use the educative component, can be idealized and applied to improve the quality of the service provided to women who seek to prevent CC, thus reducing the morbidity and mortality rates due to this disease.

This research came with some limitations. In the first place, the fact that the return appointment was not guaranteed to the women at the place of study, despite being a right, influenced the outcome in all groups. Another important aspect was that, although unintentionally, the educative group had its return appointment scheduled within a shorter interval than the other groups. In addition, the choice of the recall ribbon is also highlighted, because the material was fragile and could tear, which could compromise the objective of the intervention. Its use was also inconvenient, as it aroused other people's curiosity, which ended up being a constraint for the women.

As a suggestion for further research, a health service could be used where the return appointment is guaranteed to the women, permitting blinding for the return assessment. Another suggestion is to test other types of behavioral interventions that provide reinforcement in a shorter interval, such as a phone call or text message via mobile phone some days before the return appointment.

In addition, social interventions should be tested that use the community health agents and mixed interventions to identify the most appropriate approach to encourage the women to attend the return appointment.

## Conclusion

The women in the educative group returned to a greater extent (82%) and earlier for the appointment, confirming the superiority of the educative intervention when compared to the others. The behavioral intervention used (recall ribbon) was the least effective to reduce the rates of non-return, considering that the women in that group obtained the lowest attendance percentages of the return appointment, and within a longer interval when compared to the other groups.
